# Defining and identifying the critical elements of operational readiness for public health emergency events: a rapid scoping review

**DOI:** 10.1136/bmjgh-2023-014379

**Published:** 2024-08-29

**Authors:** René English, Heather Carlson, Heike Geduld, Juliet Charity Yauka Nyasulu, Quinette Louw, Karina Berner, Maria Yvonne Charumbira, Michele Pappin, Michael McCaul, Conran Joseph, Nina Gobat, Linda Lucy Boulanger, Nedret Emiroglu

**Affiliations:** 1 Division of Health Systems and Public Health, Department of Global Health, Stellenbosch University Faculty of Medicine and Health Sciences, Cape Town, South Africa; 2 Country Readiness and Strengthening Department, World Health Emergencies Programme, World Health Organization, Geneva, Switzerland; 3 Department of Family and Emergency Medicine, Faculty of Medicine and Health Sciences, Stellenbosch University Division of Emergency Medicine, Stellenbosch, South Africa; 4 Division of Physiotherapy, Department of Health and Rehabilitation Sciences, Stellenbosch University Faculty of Medicine and Health Sciences, Cape Town, South Africa; 5 Centre for Evidence-based Health Care, Division of Epidemiology and Biostatistics, Department of Global Health, Stellenbosch University, Cape town, South Africa

**Keywords:** Public Health, Review

## Abstract

**Introduction:**

COVID-19 showed that countries must strengthen their operational readiness (OPR) capabilities to respond to an imminent pandemic threat rapidly and proactively. We conducted a rapid scoping evidence review to understand the definition and critical elements of OPR against five core sub-systems of a new framework to strengthen the global architecture for Health Emergency Preparedness Response and Resilience (HEPR).

**Methods:**

We searched MEDLINE, Embase, and Web of Science, targeted repositories, websites, and grey literature databases for publications between 1 January 2010 and 29 September 2021 in English, German, French or Afrikaans. Included sources were of any study design, reporting OPR, defined as immediate actions taken in the presence of an imminent threat, from groups who led or responded to a specified health emergency. We used prespecified and tested methods to screen and select sources, extract data, assess credibility and analyse results against the HEPR framework.

**Results:**

Of 7005 sources reviewed, 79 met the eligibility criteria, including 54 peer-reviewed publications. The majority were descriptive reports (28%) and qualitative analyses (30%) from early stages of the COVID-19 pandemic. Definitions of OPR varied while nine articles explicitly used the term ‘readiness’, others classified OPR as part of preparedness or response. Applying our working OPR definition across all sources, we identified OPR actions within all five HEPR subsystems. These included resource prepositioning for early detection, data sharing, tailored communication and interventions, augmented staffing, timely supply procurement, availability and strategic dissemination of medical countermeasures, leadership, comprehensive risk assessment and resource allocation supported by relevant legislation. We identified gaps related to OPR for research and technology-enabled manufacturing platforms.

**Conclusions:**

OPR is in an early stage of adoption. Establishing a consistent and explicit framework for OPRs within the context of existing global legal and policy frameworks can foster coherence and guide evidence-based policy and practice improvements in health emergency management.

**WHAT IS ALREADY KNOWN ON THIS TOPIC**

- Operational readiness (OPR) has emerged as a crucial but relatively unexplored concept in the context of health emergencies.

**WHAT THIS STUDY ADDS**

- OPR is in an early stage of adoption with variable understandings of what it entails. This study highlights a need for conceptual clarity and consistency in describing OPR to build a coherent body of evidence that can underpin policy and practice. Key OPR actions aligned with five core subsystems of Health Emergency Preparedness Response and Resilience (a global, integrated framework for health emergency management) are identified.

**HOW THIS STUDY MIGHT AFFECT RESEARCH, PRACTICE OR POLICY**

- Instruments to evaluate country-level preparedness under the International Health Regulations require evidence of readiness planning. The most recent global policy framework to strengthen the global architecture for health emergencies also signposts the critical role of readiness. This scoping review has provided a foundation for global expert deliberations and agreement on OPR, which is an important step forward towards a coherent body of evidence and to advance policy and practice for improved health emergency management.

## Introduction

 A key lesson learnt from the global and national response to COVID-19 is the critical importance of early action. COVID-19 caught many countries off guard, and the consequences of delayed responses were severe in terms of public health as well as socioeconomic impacts. To prevent and mitigate the impact of future events, countries must strengthen their capabilities for rapid mobilisation to proactively respond in anticipation of an imminent threat. To this end, operational readiness (OPR) has emerged as an important part of efforts to strengthen the global architecture for health emergency preparedness, response and resilience (HEPR).^1^ HEPR, WHO’s new strategic framework, is intended to guide, inform and resource collective efforts to strengthen the key interlinked national, regional and global multisectoral capacities sitting at the intersection of health security, primary healthcare and health promotion.

In the context of the health emergency cycle, OPR arises at the intersection between preparedness planning and response.^2^ By promptly mobilising specific resources and strategies in the face of a high-priority and imminent threat, countries can enhance their ability to respond swiftly and efficiently by strategic deployment of well-defined capabilities, plans and actions that are tailored to the specific threat. The importance of this neglected phase in the health emergency cycle has catalysed related global policy initiatives. Instruments to evaluate country preparedness for emergency response under the International Health Regulations (IHR) require evaluation of country-level OPR planning, as seen in the Joint External Evaluation (JEE) 3.0’s Health Emergency Management Capacity, which targets risk-based plans for readiness and existence of an emergency readiness assessment.^3^ The WHO’s proposals for a strengthened HEPR architecture across core domains of governance, finance and systems require OPR and capacities in five core subsystems: Collaborative Surveillance; Community Protection; Safe and Scalable Care; Access to Countermeasures and Emergency Coordination, along with OPR plans in Emergency Coordination.^1 4^ Currently, there is no WHO guidance related to standardised emergency readiness assessments and readiness planning. To achieve the promise of strengthened OPR policy and practice, closer specification is needed to define what OPR involves and how it works, and the methodologies and approaches used to implement and operationalise it.

To underpin WHO technical products for OPR, we conducted a rapid scoping evidence review to examine the definitions and critical elements of OPR for public health emergencies caused by new or re-emerging infectious diseases and other public health threats in the context of the latest global policy frameworks for health emergency management.^5^ This review is important given the absence of a standardised checklist of ‘must haves’ to inform the development of a country contingency plan in the face of an emergency.

## Methods

Objectives of our review were (a) to identify how OPR has been conceptualised and defined; (b) to elicit critical elements of ‘OPRs’ in the context of key global policy frameworks, such as the WHO Global Health Security Framework, HEPR and JEE 3.0.^3 4 6^ Anticipating a large and diverse body of evidence and given the need for a rapid output from this work, we conducted a rapid, scoping review following well-recognised methods.^7^,^9^ We used the Preferred Reporting Items for Systematic Reviews and Meta-Analyses Extension for Scoping Reviews^10^ checklist for reporting. Our study protocol is published^5^ and registered (doi:10.17605/OSF.IO/6SYAH).

### Eligibility criteria

We included articles that:

1. Reported on OPRs, defined for this review, as those immediate action(s) required to preposition response actions to acute, proximal or imminent hazards and/or threats (eg, an infectious disease outbreak or a natural disaster threat), that is, an all-hazards approach^5^ in the context of health emergencies, that is, disasters and major incidents (natural and otherwise) including emerging and re-emerging infectious disease threats with the potential to significantly impact a population’s health; and described actions of emergency response groups or organisations at national, regional or global levels.
2. Types: English, German, French or Afrikaans language peer-reviewed original articles or reviews published between 1 January 2010 and 29 September 2021, publicly available policy frameworks and programme reports, published conference reports or electronic theses, relevant grey literature and documents for which full texts or abstracts were available.

We excluded articles that:

1. Focused exclusively on longer-term preparedness actions (ie, an imminent threat was not explicitly defined) or response actions (ie, actions to respond to an active public health emergency), reported on contexts beyond health emergencies or did not focus on disease prevention and control.

### Search strategy

We developed and ran a search structured by population (health systems/community), concept (readiness/preparedness/risk/planning) and context (emergencies/diseases/natural disasters) in MEDLINE, Embase and Web of Science databases (see online supplemental table S1 for detailed search strategies for the electronic databases). We searched various targeted repositories, websites and databases for grey literature^11^ (see online supplemental box S1). We also used forward and backward citation tracking.

### Selection of sources

Search outcomes were imported into Rayyan V.0.1.0 software (Rayyan Systems, Massachusetts, USA) for screening, checking of duplicates and final selection.^8 12^ Our approach to citation screening aimed to balance rigour and speed, consistent with rapid reviews and adapted from the Cochrane Rapid Reviews Methods Group’s guidance for rapid reviews,^8^ including guidance on addressing the methodological challenges faced during COVID-19 rapid reviews.^9^

Screening occurred at three levels (title, abstract and full report). The review team agreed on screening decisions upfront and agreed on guidelines after piloting for consistency.^8 13^ For piloting, two reviewers (MP and MYC) independently and in duplicate screened 100 titles and abstracts, followed by discussion with three senior authors (RE, QL and MM) to refine screening decisions. Category coding by study design and keywords for excluded articles at the title and abstract level were agreed and set in Rayyan.

After this, one reviewer (MP) screened 20% of the initially identified titles and screened abstracts to remove irrelevant reports. A second reviewer (MYC) verified excluded titles and abstracts.^8^ Conflicts and uncertainties were resolved by discussion with senior authors (RE, HG or QL). To ensure that all texts could be assessed in detail against the eligibility criteria within the limited time frame of the rapid review,^9^ full-text screening was independently conducted by eight reviewers (MYC, MP, KB, JCYN, CJ, QL, RE and HG) with the yield divided among them. Discrepancies were resolved through discussion.

### Selection of grey literature

Grey literature search outputs were screened at two levels (title and body of the report) and recorded by one reviewer (MP). A second content expert (HG) verified the included sample.^8 9^

### Data extraction and management

Two reviewers (MYC and QL) extracted data from journal articles, and one (MP) from grey literature; an additional reviewer (RE) checked for accuracy in both instances.^14^ Data were deductively coded in ATLAS.ti V.9 (Scientific Software Development) (https://atlasti.com/) and extracted into a custom-built, pilot-tested MS Excel spreadsheet, according to preset criteria The data extraction form was revised after pilot testing and consultation with WHO and amended^14^ to reflect the study authors’ affiliations and the WHO region in which the study was conducted. Uncertainties were discussed by the full review team. It was not necessary to contact the study authors.

### Appraisal

Credibility of evidence in the included articles was assessed based on the information source and type.^8^,^10^ Two reviewers (MP and MYC) appraised the included sources for descriptive purposes and incorporated the results narratively in the reflective summaries of the charting findings.

### Data analysis and presentation

To analyse data, we (QL, RE, HG, CJ and JCYN) used qualitative thematic analysis with deductive synthesis,^15^,^17^ against the following preidentified thematic categories: leadership, governance and coordination; country risk assessment; operational planning and coordination; contingency finance; health facility capacity and service delivery; health workforce/human resources; early warning or surveillance and health information systems; community resilience and risk communications; logistics or supply chain for access to essential medicines; WHO readiness and partner readiness. New themes were also identified. A revision of this analysis (HC and NG) used the new HEPR global architecture as an organising frame.^4^

### Patient and public involvement

As this study presents a scoping review of already published literature, patient and public involvement was not applicable.

## Results

**Figure 1:**
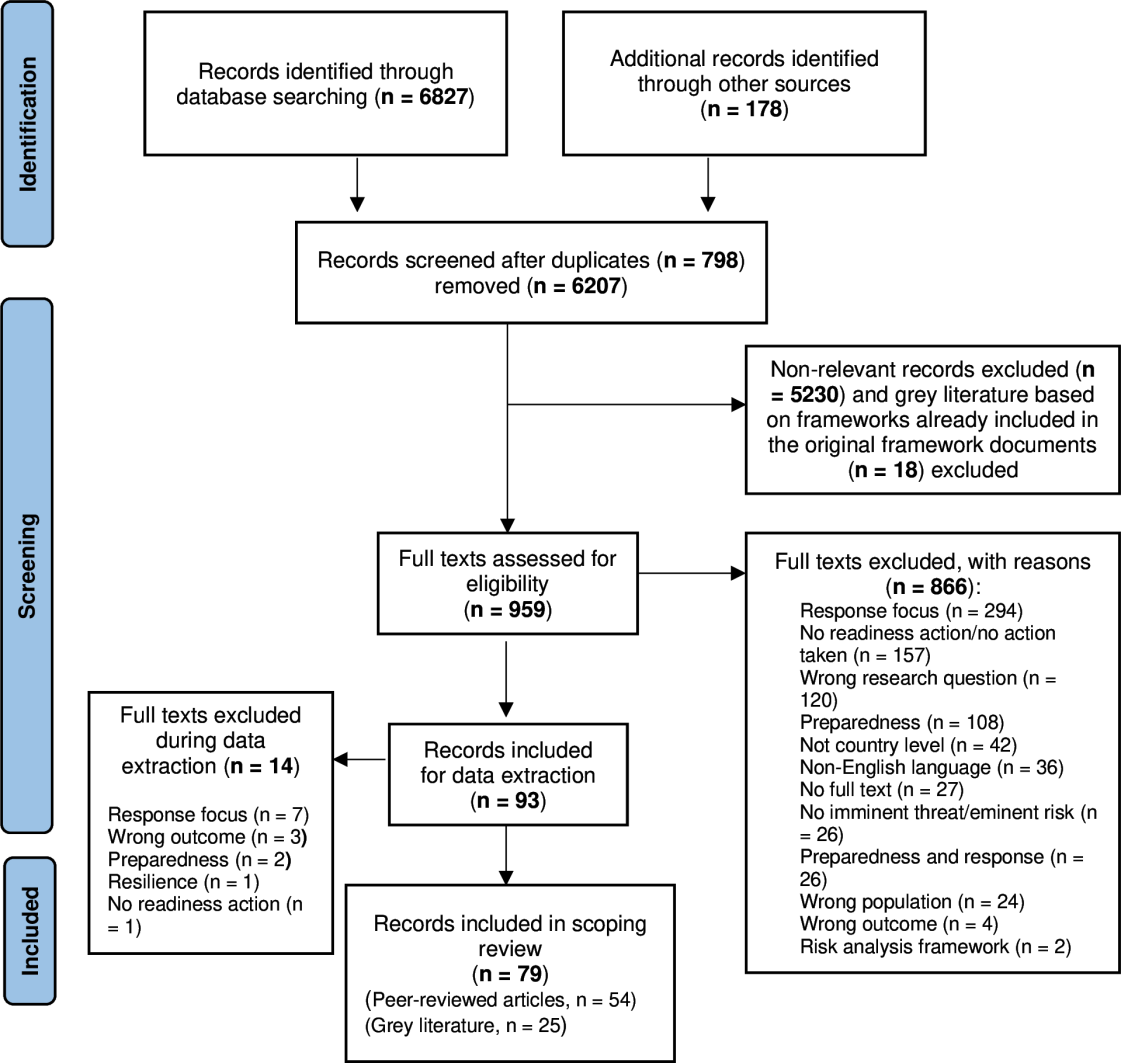
PRISMA flow diagram. PRISMA, Preferred Reporting Items for Systematic Reviews and Meta-Analyses Extension for Scoping Reviews.

Of 7005 citations identified in the database (n=6827) and grey literature (n=178) searches, we included 78 (54 peer-reviewed publications; 25 grey literature) (figure 1). The study characteristics are highlighted in onlinesupplemental table 2A B.

Online supplemental table S2A characteristics of peer-reviewed studies on the definitions of OPR according to emergency type.

Online supplemental table S2B characteristics of grey literature publications on the definitions of OPR according to emergency type.

### Definitions of OPR

Descriptions of OPR lacked clarity and consistency in definition and use. Nine primary research papers and one grey literature document provided explicit definitions of ‘readiness’ and/or ‘preparedness’ for infectious disease emergencies (online supplemental table S3).^18^,^27^ Of these, three^18 21 24^ explicitly defined ‘readiness’ while the others used the term ‘preparedness’ in a way that was congruent with our working definition of OPR. The term readiness was used interchangeably with concepts of preparedness, response and recovery. In other included articles, the concept of readiness was reflected implicitly, as per our working definition.

Some included articles suggested that preparedness indicators, using tools like the State Party Self-Assessment Annual reporting tool (SPAR), could be used to indicate gaps for the purposes of targeting OPR actions.^24 28^ Others suggested that a country’s OPR and response capacity depends on the strengths of its preparedness, with regular testing and updating of plans and capacities assessing country OPR.^22 26^ However, some authors noted that countries’ responses to COVID-19 highlighted an incongruence between IHR compliance scores and response performance; for example, some countries with lower IHR scores demonstrated a better ability to contain COVID-19 at the early stages of the pandemic.^21 29^ A lack of recently updated and tested plans and a lack of large-scale training and refresher courses or key actions for OPR, have been identified as reasons for inconsistency and weakness in previous responses.^23 25^ Others have identified the activities they had taken as a result of lessons learned from similar diseases as a reason for more successful responses.^29 30^ For example, rapid training and simulation exercises and leveraging specific expertise and experiences were considered important in preventing or mitigating an outbreak.^20 28 31^

The nature of the imminent threat also influenced the scale and speed of OPR actions, along with the proximity to the hazard.^18^,^21^ OPR could thus be considered the ‘operationalisation’ of hazard-specific capacities aimed at mitigation of a specific, identified risk. Triggering rapid action in response to an imminent threat was noted as a way to feedback and strengthen country capacities while effectively cutting costs of ‘firefighting’ public health emergencies.

While preparedness and OPR are used interchangeably by papers during this review, the reasonable abundance of literature dedicated to time-bound actions right before an event suggests they are different concepts. This observation prompts the necessity for a clear understanding of OPR and its differences from preparedness. Thus, OPR actions could build on overall preparedness levels but consist of time-sensitive activities focused on the imminent threat (eg, ensuring that the healthcare workforce has been recently trained for an imminent threat). These activities have been focused on ensuring that overall preparedness gaps are accounted for (eg, requesting international emergency medical teams (EMTs) to be ready to deploy if EMTs are unavailable in-country). In the following section, we detail the variety of OPR actions that have been taken in articles included in our review, in alignment with the HEPR subsystems.^20 28 31^

### Critical capabilities for OPR

#### Collaborative surveillance

Previous emergencies highlighted the importance of a strong Early Warning System with capacity to improve disease outbreak detection for early action to localised health events.^32^,^34^ Strong surveillance systems at all levels, rapid feedback of results and accessibility of information were described as critical for risk management and decision-making.^33 35 36^ A critical review of epidemiological data linked with planning and decision-making to increase vigilance and real-time information sharing at all levels was viewed as critical to communicate changes in the incidence of disease, which could signal triggers.^2235 37^,^40^

Key OPR actions embedded in surveillance systems included updating case definitions for consistency in identifying and reporting cases, early investigation, proactive contract tracing training for all staff and rapidly updating guidance for clinicians.^1819 23 41^,^44^ Measures to rapidly ensure integration of various types of surveillance and to address gaps in information collection and sharing were noted.^19 37 40^ Integration of human and animal health surveillance systems was viewed as critical, as was the importance of interoperability of surveillance systems.^38 40^ The interconnectivity of surveillance systems has been stressed to ensure that actions taken, and information gathered in one part of the system are made aware to other parts.^22 45^ For example, it was stressed that the occurrence of viral haemorrhagic fever in animals should activate enhanced surveillance.^38^ The timely reconciliation of data from multiple sources has been noted as challenging without an escalation in trained staff, improved communication, information technology and accessibility to more remote locations.^32^ The need to have epidemic data be open and transparent for decision-making was emphasised.^46^

OPR actions taken for surveillance systems in anticipation of a disease outbreak were centred around detecting gaps and providing solutions,^19 47^ improving case detection via procurement of supplies, distribution of case definitions and the deployment of screening teams,^2844 47^,^49^ improving reporting for Integrated Disease Surveillance and Response priority diseases^28 48^ and strengthening specimen transportation and analysis.^47^,^49^ Others included increased frequency of surveillance system results^36^ and rapid delivery of updated training and mechanisms for data sharing.^28 29 50^ Existing systems were leveraged for COVID-19 as a novel disease^37^ or the private sector engaged to provide surge capacity.^31^ Other efforts centred around digitising systems to improve flexibility of use and reporting times.^32 46^ Contact tracing systems were established as OPR actions,^44 51^ along with quarantine or isolation options, screening and referral pathways in community settings and dedicated transfers for suspect cases.^44 47 52^

OPR actions for increasing diagnostics and laboratory capacity for surveillance included prepositioning laboratory supplies in high-risk areas which was described as key to facilitating the investigation of suspected cases (eg, specimen transportation containers, triple packages and gloves, transportation vehicles for specimens).^18 19^ Electronic systems developed to improve laboratory results turnaround time,^19^ the quick detection of hotspots^36 37^ or digital contract tracing applications^37^ were important developments implemented by countries by way of OPR actions. Lessons learnt from the digitalisation of contact tracing highlighted the importance of scaling up laboratory capacity to account for the increased demand for testing and to timeously ensure sufficient capability to test and process tests.^29 31 53 54^ Mechanisms, if not available, should be rapidly instituted for sharing laboratory investigation data and establishing laboratory networks within and outside countries for timely diagnoses.^18 38^

Included sources also signposted OPR actions for a collaborative approach to successful surveillance. For the rapid confirmation of novel influenza strains, for example, countries were successful in collaborating with WHO collaborating centres in their region.^35^ Laboratory capacity in other countries were rapidly increased through the creation of laboratory networks.^18 42^ In scenarios where a neighbouring country had a disease outbreak, cross-border surveillance teams have been established and the sharing of information between border countries improved and highlighted as a reason for the limited spill.^19^ During COVID-19, surveillance was rapidly readied at the point of entries, including standard operating procedures for detected cases and awareness-raising sessions for personnel.^55 56^

#### Community protection

Included articles highlighted key actions to upscale for rapidly involving and engaging affected communities in anticipation of an imminent threat.^22 57^ These include rapidly providing updated information about the threat, including on identifying symptoms and any known public health and social measures, disseminated through numerous mechanisms and in a variety of languages to those at risk.^19 23 31 33 46^ These should be adapted for all literacy levels.^58^ Value was found in daily communications to build public trust.^37^ Community volunteers were trained to carry out communal and door-to-door health education^19 32^ or public websites containing epidemic reports to keep communities informed.^46^

Further recommendations highlighted risk communications and public health and social measures to be rapidly readied to contain any potential community transmission.^1821 48 51 59^,^61^ These communications should allow the public to have a proper understanding of the perceived risk.^35^ Other recommendations included working with local influencers to disseminate trusted information^47^ and creating specialised focus messages for high-risk populations.^26 62^ Crucially, there should be strong efforts for engaging vulnerable populations.^28 31 57^

Plans and protocols should be in place for community-specific risk assessments to fill gaps in community OPR.^28^ These assessments should focus on community perception, knowledge, preferred and accessible communication channels and existing barriers preventing community members from adopting promoted behaviours.^47^ Plans should further account for resources for social security to support vulnerable communities.^40^ To support this, community-based measures such as leveraging the community health workforce and community-based actors should be considered.^52^ In this way, community needs and realities can be accounted for in the development of risk communication and community protection interventions. Misconceptions in the community should be identified and efforts made to dispel misinformation.^44^

Some papers highlighted the early identification of vulnerable and remote population groups to ensure that their unique needs are well understood and addressed both in the design of interventions and in mitigating the impact of response interventions.^28 57^ Accordingly, planning OPR should involve the input of communities, particularly organisations representing vulnerable groups, to inform community OPR.^47^ Plans for response action should additionally consider secondary impacts or unintended consequences. For example, a clear lesson from COVID-19 related to the need for social security policies to mitigate the impacts of restrictive public health and social measures.^63^ Policies for implementation should incorporate social security safety nets for communities, such as social health protection schemes or providing financial assistance for quarantined populations.^40^ Plans should further be supported by partners.^64^ Indirect health impacts should also be considered when OPR actions are implemented.^65^ For example, some countries rapidly scaled up their capabilities for mental health services by implementing psychiatric hotlines^66^ or providing stress management protocols.^48^ Other indirect health impacts could include food insecurity; to prevent this, doorstep delivery of daily essentials^31^ or provision of prepackaged meals^39^ were planned.

Numerous papers highlighted the need for public health and social measures to be available rapidly and as early as possible, such as (for respiratory disease outbreaks) mask usage in public places when the risk level was high^31 36 46 52 63^ and access to water, sanitation and hygiene,^44 48^ with additional measures in place for individuals at risk of complications at the household level, such as using physical barriers, proper wearing of masks and environmental cleaning.^52^ If non-existent, a strategy should be in place to assist in accelerating the containment of disease through imposing various public health and social measures, such as limits on local and international travel, the wearing of masks in public places,^37^ social distancing,^67^ bans or limits on mass gathering events^33 48^ and closing educational institutions.^36 48^ These measures were all implemented to a varying degree during COVID-19, with analyses finding that the earlier efforts of containment generally resulted in better containment early in the pandemic.^21^ The measures taken should be weighed against the possibility of improving detection and spread through other methods, such as a rapid expansion of laboratory testing.^63^ Public health and social measures should additionally take into account other likely risks—for example, countries with hurricane-prone areas during COVID-19 had to quickly revise their strategies to ensure social distancing in shelters.^39^ If vaccines are available, a prioritisation policy should be developed to avoid ethical and political conflicts.^23^

#### Safe and scalable care

For the health service to function during an emergency, they need a baseline quota of adequate staffing to perform core functions.^68^ Included articles stressed OPR to surge additional healthcare personnel.^31^ The healthcare workforce needs updated case definitions, transmission, clinical presentation, infection prevention and control (IPC), community surveillance and case management for the threat.^19^ Capacity assessments can guide OPR to estimate the ability of health systems to contain the imminent threat^36 37 41^ and to identify gaps.^29 36^ Additional recommendations highlight that capacity modelling should integrate risks to the workforce during the response—previously, health workforce absenteeism has not always been considered in the development of staffing plans, leading to reduced response capacities.^57^ When scaling up healthcare worker OPR for a threat, actions should also be taken to scale up the services to support them.^64^ Health systems gaps have been addressed by increasing the space of intensive care unit beds in relevant facilities, human resource training and mobilisation^2036 48 49 63 69^,^71^ and reducing the workload (eg, patients with mild symptoms were managed at home in isolation).^46^ Referral systems and safe pathways should be established.^36 42 52^

COVID-19 highlighted the importance of maintaining essential health services during an emergency. Many studies under review did not immediately prioritise this when considering OPR for the imminent threat. Measures taken proactively to maintain essential health services and to reduce the stress on the health system were described, such as giving patients with chronic diseases a stockpile to prevent them from coming to the hospitals^31 72^ and use of telemedicine.^31 40^ It was recommended to establish referral systems and safe pathways to designated local isolation facilities and enhance case detection in healthcare facilities and the community.^47^ Others emphasised their learnings from response to diseases before COVID-19 and maintaining the continuum of care^36 40^- for example, Korea created two systems (COVID-19 health system vs non-COVID-19 health system) to ensure continuity of non-COVID-19 needs and diverted the flow of patients through triage centres.^36^ Measures were taken to safeguard hospitals not identified as part of the response, for example, using temperature checks or encouraging the use of masks.^33 40^

Included articles also noted that staff protection and welfare should be strongly included in OPR planning, for example, to anticipate provision of personal protective equipment (PPE) and supplies for staff protection.^73 74^ An IPC programme should be implemented before an outbreak.^33 38 63^ Prepositioning of PPE supplies in high-risk districts has been recommended to enable a more rapid response,^19^ or if the risk level is low, the availability of a regional reserve of PPE.^75^ Where PPE was unavailable, production was quickly ramped up to be able to maintain inventory before the response^76^ - others who did not do this noted that they suffered shortages during the response.^46^ Regular training and simulation exercises were conducted for case management teams.^19 38^ Psychosocial support and other interventions necessary to support staff welfare were also emphasised.^26 40^ Others quickly put legislation into place to protect healthcare workers engaged in response from being attacked.^31^

#### Access to countermeasures

There were fewer descriptions of OPR in this HEPR subsystem in comparison with others. When gearing up for response, countries have increased production and procurement by procuring from local industry, working with manufacturing companies to increase supply by, for example, adapting manufacturing facilities or establishing warehouses and transportation.^18 31^ Numerous studies noted that they had extreme difficulty in obtaining the supplies they needed,^40 46^ due to limited stockpiles and lack of finances to maintain them.^23^ OPR actions for an imminent threat would focus on scaling up manufacturing plans and to ensure that a stockpile is in place.

Prepositioning essential supplies is essential for OPR, with an adequate supply of medical equipment to the frontline identified as vital for reducing health emergency risks.^77^ Additionally, measures to quickly acquire and distribute medical supplies using government-set prices, prioritise frontline health professionals and vulnerable populations for the disbursement of medical countermeasures and promote local manufacturing were identified.^20^ Other countries described OPR actions to introduce therapeutics, diagnostics and vaccines.^37^

One study identified research topics such as system OPR, knowledge, attitudes and practices of the health workforce, epidemiology of the disease at the national level, best practices at the points of entries and isolation centres and infection-control measures as important to inform OPR actions.^78^ Research should also support decision-making, cost-effectiveness, intervention effectiveness and the impact of these on pandemic trajectories.^50 79^ Competing demands can limit the volume of research conducted which was considered a missed opportunity.^32^ Early convening of expert groups to advise government was identified as useful for managing health service responses and OPR, and their work should as far as possible be informed by evidence (eg, scenario planning).^33^ Health systems researchers occupying the highest levels of oversight across the sectors were said to enhance the use of evidence and data for decision-making.^36^ Another paper noted that lessons learnt by regions found that funding for research and investigations during OPR and response should also be in place.^39^

#### Emergency coordination

We identified several critical and overarching governance-related elements that facilitated OPR within regions and countries. Lessons from OPR or responses to previous diseases have demonstrated the importance of a coordinating body at regional or national levels^19 35 36 41 42 46 48 75 78 80 81^ led by high-level officials.^19 48 80^ These structures should provide leadership and coordination,^42 46 62 82^ guidance and action plans,^36^ and communication of critical information.^48 80^ Strong and skilled leadership was a notable enabler^29 32 36 54 83^ and was marked by active OPR involvement of the responsible health departments, and effective coordination with multiple stakeholders as the planning or response evolved.^29 32 54 82 84^ Flexibility and adaptation, particularly during OPR, were important.^32^

Many included articles emphasised the timely activation of coordination mechanisms and risk assessments to inform plans.^18 19 31 34 38 47 54 69 75 83 85 86^ This involved the establishment and operationalisation of intersectoral and/or interdisciplinary teams (eg, task teams,^19 33 75 80^ special councils^41 42 46^ and command centres^30 41^) to provide technical expertise,^25 42 78 87^ prepare and coordinate the implementation of policy decisions^32 80 87^ and guide lower health system-level or governmental-level structures or actors.^28 32 88^ An Incident Management System was adopted in several countries with a dedicated lead,^32 35 36 83 89^ and this was further recommended in the grey literature.^72 90 91^ When operationalising these aspects for an efficient and effective response, the early establishment of clear roles and responsibilities, with a clear lead was considered vital and instrumental for later response success.^28 32^ The highest levels of government should be involved, with an all-of-society and/or all-of-government approach.^3235 69 70 79 87^,^89^

To successfully implement coordination and response to an emergency, workforce management is key for a successful response. Actions taken include recruitment of staff from the private sector, healthcare students or retired or non-practising trained workers,^31 40 42 48 78 89 92^ community health workers and community-based organisations^19 31 40 48 73^ or volunteers.^19 48 89^ Grey literature emphasised, actions in support of cross-border response teams or surge teams with rapid staff registration and accreditation systems, staff redeployment and reallocation,^18 72 93^ and appropriate training.^18 90 94 95^ Also critical was ensuring the availability of emergency medical services for immediate response and the early deployment of multidisciplinary Rapid Response Teams in high-risk groups.^23 31 53 83 87 89^ Some papers emphasised prioritising actions which enable rapid deployment of these teams.^53 83^

Other important factors included threat-specific contingency planning at national and subnational levels for identifying preparedness gaps and actions to work around them, thus supporting rapid detection, response and containment.^18 19 35 83 89^ Contingency plans helped to prioritise targeted actions^83^ as well as identify and prioritise at-risk geographic areas and vulnerable communities.^40 57^ Having recently updated or tested contingency plans in place was stated as essential to enhance OPR and effective response,^25 39 96^ and these should support operations and logistics, help understand organisational structures and functions, and optimise resources.^44 68 93^ They should further ensure critical infrastructure for health system functioning and ensure clinical and health service-level plans are detailed and able to assist in preparing for increased patient volumes or need for critical care services.^19 68^ Contingency plans should incorporate past experiences and learnings from other outbreaks, changing contexts^52^ and the results of simulation exercises conducted on the preparedness and response systems.^18 19 23^ Countries with similar public health emergency experiences have been found to be better prepared than those without previous experience,^63^ raising the importance of practice, via simulation exercises and training, for a new imminent threat.^19 23^

Furthermore, country risk and vulnerability assessments should be available and guide risk assessment activities.^19 31 35 38 39 47 52 53 57 84^ They were recommended to be focused on geographical areas with particularly high assessed risks^39 52 89^ and related to prevention and control strategies.^19 47 84^ The assessments should be conducted to ensure that the contingency plans contain appropriate OPR actions and consider local contexts^47 68 89^ and can also be used to guide the prioritisation of actions.^47 89^ Risk assessments for future waves or outbreaks should also be conducted, and updated worst-case scenarios incorporated into contingency plans.^39 63^

OPR needs bespoke financial planning.^22 28 70^ It was recommended that contingency funds be available for OPR,^83^ ring-fenced and situated within a dedicated emergency programme.^19 50 70^ There should be existing emergency financial management systems which allow for rapid, transparent and efficient use of funding.^40 42^ Contingency funds were emphasised as particularly important as resources should not be diverted from necessary routine programmes.^25 50^ Having contingency funds in place would ensure a few key capacities: first, earmarked resources for the hazard are ensured^22^ and lead to rapid activation of key surveillance and early response activities.^25 50^ Second, changes which may need to occur to financing healthcare services are already outlined, such as creating financial protection mechanisms for discontinued outpatient services or outlining how citizens or health insurance systems pay for screening and diagnostic testing.^42^ Finally, contingency funds should cover workforce surge, including staff, supplies, training and workforce management.^73^

## Discussion

This scoping review examined definitions and critical elements of OPR for public health emergencies. We sought to identify key actions that were mobilised in anticipation of an imminent threat framed in the latest conceptualisation of a global architecture for health emergency management. From 54 peer-reviewed publications and 24 grey literature sources, we found that the concept of OPR was in an early stage of adoption. Where the term was explicitly defined, these definitions lacked coherence and consistency and included articles that matched our working definition of OPR, often did not use the term. Our analysis highlights the important need for conceptual clarity regarding OPR. We agreed on a working definition of OPR at the outset of the review as those immediate actions taken in the presence of an imminent threat that is rapidly mobilised or prepositioned to respond to that threat. It was also often difficult to identify where the line between preparedness, OPR and response lay. For our purposes, these distinctions are relevant in so far as they can guide early detection and timely activation of key OPR capabilities in useful and practical ways. Put simply: when a hurricane is coming, you may rapidly begin to take measures to prevent damage to your house. These could include actions such as securing loose objects, protecting windows, turning off utilities and filling tubs with water. These actions, taken before a storm, would differ greatly from the years spent building and maintaining the house beforehand - ensuring the foundation is sound, and the roof has been well maintained. They would further differ from the actions would take immediately during and after the storm has hit.

This review was initiated during the dynamic and fast-moving context of a pandemic where important policy developments were advancing in parallel. To maximise the utility of this work, we reanalysed our findings to map to the HEPR framework once it became publicly available for wider discussion among WHO member states. Our analysis across the body of articles included in this review identified OPR actions that mapped to the five core subsystems considered critical to strengthen the global HEPR. Additionally, our review mostly identified national-level capabilities and provided less insight into key actions to activate subnational and local capabilities. This observation may reflect a limitation of our review, an under-reporting, or a need to further develop and define OPR at these levels.

Across articles included in this review, OPR actions were identified as those that aimed to fill gaps in a country’s capacities or to prepare for an early response. In this way, a key contribution of embedding OPR in health emergency management is in institutionalising prompt action as soon as a potential signal is detected. Of note are the many actions identified for emergency coordination, including strong, high-level leadership, governance and coordination, with clarity around the roles and responsibilities of the leaders and the coordination bodies. Collaborative surveillance that allows for early detection of signals is key for OPR in terms of triggering action. This is an underdeveloped part of readiness practice. Other important areas included rapid, integrated and interoperative health information systems for purposes such as surveillance, planning and decision-making, managing operations, and monitoring country responses. The ability to rapidly plan for, mobilise and manage resources (eg, human, PPE, financial) and scale-up services (eg, essential or laboratory) underpinned by supportive legislation were also identified. Clear and strong communication at the level of the policy-maker, within the services and in the community was also identified as crucial for optimal OPR. We note gaps related to research and manufacturing platforms enabled by technology and our analysis did not consider OPR actions at the intersection of the five subsystems, for example, the readiness of communities for early detection to support collaborative surveillance or for participation in clinical trials of novel medical countermeasures.

The review methodology has strengths and limitations. This work was done rapidly by a large team with the aim of underpinning practical technical products for OPR in health emergency risk management. A scoping review methodology was best suited to answer our research question, due to the broad base of evidence.^13^ As far as possible, we followed expert group recommendations on the adaptations needed in the conduct of rapid reviews.^9 14^ Our initial analysis mapped key thematic categories in the HEPR Framework.^1^ To align with global policy developments that have led to the HEPR framework, we updated our analysis. In this process, we may have missed new articles that would add further insight into OPR experience. However, given the pragmatic focus for this review, and the global consensus work that has followed, it is unlikely that further updates to this review would significantly alter our key conclusions. Since this review, there has been significant progress in actions to strengthen the global HEPR architecture. A more thorough review of OPR, one for each of the subsystems, is needed to reflect the recently published breakdown of HEPR subsystems into capabilities.^4^ Further, as OPR becomes engrained in health emergency response, a review to identify the optimal time frame needed to quickly and effectively operationalise the capabilities of every subsystem is needed. Additionally, the purpose of the review was not to identify how OPR actions have increased resilience. Future research is needed now that OPR has been defined to identify the OPR interventions which maximise populations; abilities to withstand an event and increase resilience. Finally, our review does not include a body of work on anticipatory actions, which aligns well with OPR. Anticipatory actions are defined as ‘actions taken ahead of predicted hazards to prevent or reduce acute humanitarian impacts before they fully unfold’.^97^ They highlight OPR as part of emergency management, particularly for disaster management and in humanitarian contexts.^98^ The outcome of these meetings reflects a growing consensus on the critical importance of OPR. The essence of OPR is to mobilise early action when a threat is on the horizon. The work reported in this paper is an important step to advancing this important and urgent agenda. Indeed, this work has now set a foundation for the more substantive and coherent development of the evidence in this important area and has provided input to readiness actions within the recently published IHR Benchmarks, and is informing the creation of readiness assessments and has informed the creation of a readiness course on OpenWHO.^99 100^

## Supplementary material

10.1136/bmjgh-2023-014379 online supplemental file 1

10.1136/bmjgh-2023-014379 online supplemental file 2

10.1136/bmjgh-2023-014379 online supplemental file 3

10.1136/bmjgh-2023-014379 online supplemental file 4

10.1136/bmjgh-2023-014379 online supplemental file 5

## Data Availability

Data are available on reasonable request.
