# Supplementary material for: Defining and identifying the critical elements of operational readiness for public health emergency events: a rapid scoping review
Source: BMJ Glob Health. 2024 Aug 29;9(8):e014379. doi: 10.1136/bmjgh-2023-014379 (PMC11367384; doi:10.1136/bmjgh-2023-014379)
Supplement: online supplemental file 1 [file bmjgh-9-8-s001.pdf]

Supplemental Box S1: Repositories, websites, and databases searched to identify grey literature sources

Global organisations, e.g.:

- World Health Organisation (WHO), United Nation Children's Fund (UNICEF), United Nations Office for Disaster Risk Reduction (UNDRR), United Nations International Strategy for Disaster Reduction (UNISDR), International Federation of Red Cross (IFRC), International Committee of the Red Cross (ICRC)

Regional WHO offices, i.e.:

- Southeast Asian, African, Western Pacific, Pan American, European and Eastern Mediterranean

European Centre for Disaster Medicine (CEMEC)

Societies and organisations, i.e.:

- World Association for Disaster and Emergency Medicine (WADEM), Médecins Sans Frontières (MSF), ReliefWeb

National websites, i.e.:

- United States Centres for Disease Control and Prevention (CDC) and Federal Emergency Management Agency (FEMA), Robert Koch Institute (RKI), Public Health England. Lastly

Evidence repository, i.e.:

- Evidence Aid
