# Supplementary material for: Defining and identifying the critical elements of operational readiness for public health emergency events: a rapid scoping review
Source: BMJ Glob Health. 2024 Aug 29;9(8):e014379. doi: 10.1136/bmjgh-2023-014379 (PMC11367384; doi:10.1136/bmjgh-2023-014379)
Supplement: online supplemental file 2 [file bmjgh-9-8-s002.pdf]

Supplemental Table S1: Detailed search strategies for the electronic databases

Database	Query		Records retrieved
**MEDLINE**	**Ovid MEDLINE(R) and In-Process, In-Data-Review & Other Non-Indexed Citations <1946 to September 27, 2021>**		
	1	Disease Outbreaks/	85271
	2	epidemics/ or pandemics/	77571
	3	(catastrophe or disaster or drought or earthquake or evacuation or famine or flood or floods or hurricane or cyclone or landslide or landslide or tsunami or tidal wave or volcano).tw.	95527
	4	Natural Disasters/	348
	5	Public health emergency.mp.	2882
	6	COVID-19/	105511
	7	Covid.tw.	132306
	8	Hemorrhagic Fever, Ebola/	6009
	9	Ebola virus.mp. or Ebolavirus/	6362
	10	SARS Virus/ or SARS-CoV-2/ or SARS.mp.	113897
	11	yellow fever.tw. or Yellow Fever/	7064
	12	Influenza, Human/	52855
	13	Communicable Diseases, Emerging/	6305
	14	emerging virus.mp.	1360
	15	1 or 2 or 3 or 4 or 5 or 6 or 7 or 8 or 9 or 10 or 11 or 12 or 13 or 14	407332
	16	Disaster Planning/ or preparedness.tw.	27006
	17	readiness.tw.	17895
	18	risk assessment.tw. or Risk Assessment/	322294
	19	Disaster Medicine/ or "Emergency Medical Services"/	33078
	20	public health response.mp.	1632
	21	emergency planning.mp.	501
	22	disaster management.mp.	1370
	23	operational readiness.mp.	169
	24	Leadership/	43759
	25	governance.tw.	14287
	26	Incident Management System.mp.	111
	27	Emergency Operations Centre.mp.	27
	28	multi-sector coordination.mp.	3
	29	Country Risk Profile.mp.	0
	30	Operational planning.mp.	174
	31	("emergency response plan" or "contingency plan").mp. [mp=title, abstract, original title, name of substance word, subject heading word, floating sub-heading word, keyword heading word, organism supplementary concept word, protocol supplementary concept word, rare disease supplementary concept word, unique identifier, synonyms]	1177
	32	(logistics and supply chain).mp. [mp=title, abstract, original title, name of substance word, subject heading word, floating sub-heading word, keyword heading word, organism supplementary concept word, protocol supplementary concept word, rare disease supplementary concept word, unique identifier, synonyms]	267

	33	Contingency finance.mp.	0
	34	Drugs, Essential/ or essential medicine.mp.	1977
	35	logistic supply.mp.	7
	36	early warning.mp.	7531
	37	Public Health Surveillance/	4719
	38	community resilience.mp.	561
	39	16 or 17 or 18 or 19 or 20 or 21 or 22 or 23 or 24 or 25 or 26 or 27 or 28 or 29 or 30 or 31 or 32 or 33 or 34 or 35 or 36 or 37 or 38	466764
	40	15 and 39	29015
	41	health system.mp.	61652
	42	health services.tw. or Health Services/	95945
	43	Public Health/	87343
	44	health facilities.mp. or Health Facilities/	29065
	45	health personnel.mp. or exp Health Personnel/	625414
	46	41 or 42 or 43 or 44 or 45	857447
	47	40 and 46	5815
	48	limit 47 to yr="2010 -Current"	4275
**Embase**	**Embase <1996 to 2021 Week 38>**		
	1	Disease Outbreak.tw.	7421
	2	epidemic/ or pandemic/	187037
	3	(catastrophe or disaster or drought or earthquake or evacuation or famine or flood or floods or hurricane or cyclone or landslide or landslide or tsunami or tidal wave or volcano).tw.	96165
	4	Public health emergency.mp.	3299
	5	natural disaster/	3548
	6	Covid.tw.	157374
	7	coronavirus disease 2019/	148801
	8	Ebola hemorrhagic fever/	6883
	9	Ebolavirus/	3761
	10	SARS.mp. or severe acute respiratory syndrome/	80752
	11	yellow fever/	3932
	12	influenza/	25733
	13	Communicable Diseases, Emerging/	12560
	14	1 or 2 or 3 or 4 or 5 or 6 or 7 or 8 or 9 or 10 or 11 or 12 or 13	442828
	15	Disaster Planning/ or preparedness.tw.	26939
	16	readiness.tw.	21708
	17	risk assessment.tw. or Risk Assessment/	630413
	18	public health response.mp.	1779
	19	emergency planning.mp.	656
	20	disaster management.mp.	1674
	21	operational readiness.mp.	202
	22	Leadership/	70482
	23	governance.tw.	18071
	24	Incident Management System.mp.	154
	25	Emergency Operations Centre.mp.	36
	26	multi-sector coordination.mp.	6
	27	Country Risk Profile.mp.	0
	28	Operational planning.mp.	204
	29	("emergency response plan" or "contingency plan").mp.	1478
	30	(logistics and supply chain).mp.	417

	31	Contingency finance.mp.	0
	32	logistic supply.mp.	19
	33	early warning.mp.	9804
	34	Public Health Surveillance.tw.	2351
	35	community resilience.mp.	576
	36	health system.mp.	81325
	37	health services.tw. or Health Service/	196744
	38	Public Health/	177806
	39	emergency health service/	91713
	40	disaster medicine/	1419
	41	essential drug/	1412
	42	essential medicine.tw.	2275
	43	15 or 16 or 17 or 18 or 19 or 20 or 21 or 22 or 23 or 24 or 25 or 26 or 27 or 28 or 29 or 30 or 31 or 32 or 33 or 34 or 35 or 36 or 37 or 38 or 39 or 40 or 41 or 42	860867
	44	14 and 43	42998
	45	health care facility/	62270
	46	health personnel.mp. or health care personnel/	247442
	47	28 or 29 or 30 or 46 or 47	688599
	48	44 and 47	9874
	49	limit 48 to yr="2010 -Current"	7852
	50	limit 49 to exclude medline journals	1076
**Web of Science**	**Science Citation index expanded and Social Sciences citation index (Web of Science)**		
	1	epidemic or pandemic or "natural disaster" or earthquake or evacuation or famine or flood or floods or hurricane or cyclone or landslide or landslide or tsunami or Covid-19 or Ebola or "yellow fever" or "human influenza" or "emerging diseases" (Topic)	617867
	2	preparedness or readiness or "risk assessment" or "Emergency Medical Services" or "public health response" or "emergency planning" or "disaster management" (Topic) or Leadership or governance or "Incident Management System" or "Emergency Operations " (Topic) or logistics or "supply chain" or "essential drugs" or "early warning" (Topic)	813269
	3	(#2) AND #1	38333
	4	(#2) AND #1 and 2022 or 2021 or 2020 or 2019 or 2018 or 2017 or 2016 or 2015 or 2014 or 2013 or 2012 or 2011 or 2010 (Publication Years)	33618
	5	((#2) AND #1) AND TS=("health system" or "health services" or "health facilities" or "health personnel")	1624
	6	((#2) AND #1) AND TS=("health system" or "health services" or "health facilities" or "health personnel") and 2022 or 2021 or 2020 or 2019 or 2018 or 2017 or 2016 or 2015 or 2014 or 2013 or 2012 or 2011 or 2010 (Publication Years)	1476
