# Supplementary material for: Defining and identifying the critical elements of operational readiness for public health emergency events: a rapid scoping review
Source: BMJ Glob Health. 2024 Aug 29;9(8):e014379. doi: 10.1136/bmjgh-2023-014379 (PMC11367384; doi:10.1136/bmjgh-2023-014379)
Supplement: online supplemental file 3 [file bmjgh-9-8-s003.pdf]

Table 1-A: Characteristics of peer-reviewed studies on the definitions of operational readiness according to emergency type.

**Coronavirus disease 2019 (COVID-19)**					
**Author(s) and publication year**	**Country under study (World Bank classification)**	**WHO region**	**Study design**	**Purpose (abbreviated summary)**	**Description of study participants for primary studies or data sources for secondary studies**
**PRIMARY STUDIES**					
Bennett 2021 (51)	Australia (HIC)	WPR	Descriptive report	To report on Australia's national and state public health pandemic response.	N/A
Biswas et al. 2020 (54)	Bangladesh (LMIC)	SEAR	Document analysis	To conduct a situation analysis on Bangladesh's COVID-19 pandemic preparedness.	Published reports (government, non-governmental organisations, mainstream media).
Ghanbari et al. 2020 (41)	Iran (LMIC)	EMR	Qualitative document analysis	To assess Iran's health system preparedness and COVID-19 response to the outbreak.	Relevant government documents, program reports, official statements.
GRID COVID Study Group 2020 (31)	India (LMIC)	SEAR	Document analysis	To describe India's early COVID-19 response	Relevant government documents, program reports, official statements and data, media.
Kandel et al. 2020 (24)	182 countries (Varied)	Varied	Document analysis	To analyse the 2018 health security capacities submissions of 182 related to COVID-19 outbreak.	2018 SPAR submissions
Liu & Saltman 2020 (66)	China (UMIC)	WPR	Descriptive report	To review early disease control actions taken by the Chinese central government (January 20 – 27)	N/A

Moonasar et al. 2021 (37)	South Africa (UMIC)	AFR	Descriptive report	To describe measures taken by South Africa to contain the spread and mitigate effects of COVID-19.	N/A
Neogi & Preetha 2020 (29)	Europe (Italy and Spain), Western Pacific (Australia, China, and Singapore), Southeast Asia (South Korea and India), Eastern Mediterranean (Saudi Arabia and Egypt), Americas (the USA and Brazil), and Africa (Nigeria and South Africa) (Varied)	EUR, WPR, SEAR, EMR, AMR, AFR	Document analysis	To assess the health systems strength and their capacities to respond to COVID-19 pandemic threats.	Secondary data available in the public domain.
Neupane et al. 2020 (87)	Nepal (LMIC)	SEAR	Descriptive report	To identify the key public health measures to contain COVID-19.	N/A
Nguyen et al. 2021 (71)	Vietnam (LMIC)	WPR	Descriptive report/case reports	To describe the response to and status of the COVID-19 pandemic in Vietnam.	N/A
Oh et al. 2020 (36)	The Republic of Korea - South Korea (LIC)	WPR	Descriptive report	To investigate how laws across Europe relate to	N/A

				pandemic preparedness planning.	
Oliveira et al. 2020 (79)	Brazil (UMIC)	AMR	Descriptive report	To report on the Brazilian Ministry of Health's COVID-19 actions.	N/A
Paudyal et al. 2021 (67)	Belgium, Croatia, Czech Republic, Denmark, England, Estonia, France, Republic of Ireland, Italy, Netherlands, Northern Ireland, Portugal, Serbia, Spain, Switzerland, Turkey (UMIC and HIC)	EUR	Qualitative	To explore the views and experiences of clinical pharmacists with regards to clinical pharmacy service provision during COVID-19.	Twenty-two pharmacists representing 16 European countries (in hospital, community, and primary care settings).
Petrović et al. 2020 (21)	European Countries (HIC)	EUR	Multidimensional analysis	To bringing together diverse aspects of readiness and initial reaction to a COVID-19 outbreak.	Secondary data from 23 European Countries
Raoofi et al. 2020 (48)	Iran (LMIC)	EMR	Qualitative content analysis	To compare the policies and strategies that Iran is adopting, with the experience and recommendations of China and WHO to combat COVID-19.	Available policy documents, programs, action plans, reports, websites and official news

Santos et al. 2021 (43)	Brazil (UMIC)	AMR	Policy analysis	To analyse the government's strategic agenda for coping with COVID-19 in Brazil, focusing on hospital care.	Twenty-eight Contingency Plans, one national, 26 state, one Federal District.
Shimizu & Negita 2020 (49)	Japan (HIC)	WPR	Document analysis	To clarify how Japan responded to the first wave of the COVID-19 pandemic.	Recorded epidemiological trends of COVID-19 in Japan in January–May 2020,
Simões et al. 2020 (42)	Germany, Spain, France, Italy, Portugal, and the U.K. (HICs)	EUR	Analysis of secondary epidemiological data	To analyse the response in the first months of 2020 to the SARS-CoV-2 pandemic in Germany, Spain, France, Italy, Portugal, and the U.K.	Secondary epidemiological data.
Tran et al. 2020 (77)	Vietnam (LMIC)	WPR	Cross-sectional survey	To assess the operational readiness capacities of the grassroots health system in response to epidemics	6029 respondents, most of them were medical students
Wang et al. 2020 (80)	China (UMIC)	WPR	Qualitative document analysis	To summarise the evolution of COVID-19 in China.	Relevant government documents, program reports, official statements.
Wang et al. 2020 (69)	Iran, Japan, Republic of Korea (South Korea), the U.K., and the USA	EMR, WPR, EUR, AMR	Cross-sectional document analysis	To examine the health system resilience of select countries, their strategies, and countermeasures in	Relevant government documents, program reports, official statements and data, media.

	(HIC and one LMIC (Iran))			response to the COVID-19 pandemic	
**SECONDARY STUDIES**					
Hasan et al. 2021 (75)	AFR (Tunisia, Bolivia, Algeria, Cameroon, Cote d'Ivoire, Gambia, Madagascar, Nigeria, Rwanda, Senegal, South Sudan, Uganda) and EMR (Egypt, Iraq, Jordan, Morocco, Saudi Arabia, Sudan, Tunisia), SEAR (India, Nepal), WPR (Vietnam). (LIC and LMIC)	AFR, EMR, SEAR, WPR	Systematic scoping review	To synthesise the emerging evidence on integrated health service delivery (IHSD) approaches adopted in L-LMIC during the COVID-19 pandemic.	Relevant government documents, program reports, official statements and data, media.
He et al. 2020 (46)	Belgium (HIC)	EUR	Narrative review	To describe the Belgian COVID-19 responses process Belgium.	Relevant government documents, program reports, official statements and data, media.
Mohammadpour et al. 2021 (70)	Not specified	N/A	Scoping review	To determine the factors affecting the readiness and responsiveness of healthcare	60 articles yielded from five online databases from January 2000 to June 15, 2020.

				systems during epidemic crises	
Sohrabizadeh et al. 2021 (39)	Amanda, Australia, Canada, China, Croatia, Iran, Islands, Vanuatu, Fiji, Tonga, Puerto Rica, the USA, South Asian countries, and the world (Varied).	Varied	Systematic review	To identify the literature focused on health system response to coincidence of COVID-19 and disasters.	13 published articles
Yang et al. 2021 (63)	Republic of Korea (ROK) (HIC)	WPR	Narrative review	To summarise and compare the epidemiology and response of the ROK to the 2015 MERS outbreak and the COVID-19 epidemic in early 2020.	Not mentioned
**MULTIMETHOD STUDIES**					
Kapiriri et al. 2021 (50)	Uganda (LMIC)	AFR	Qualitative and document review	To discuss how priority setting and resource allocation could be integrated into WHO pandemic planning and preparedness framework and used to inform the COVID-19 pandemic recovery	Health workers and policy makers who participated in the control of the EVD outbreak in Uganda.

				plans and plans for future outbreaks.	
Seyedin et al. 2021 (96)	Not specified	N/A	Scoping review, qualitative and checklist development	To develop a checklist for evaluating the preparedness of hospitals to respond to the COVID-19 pandemic.	Relevant articles from January 2019 to April 2020 Participants included experts from multiple disciplines
**OPINION STUDIES**					
Al Nsour et al. 2020 (78)	Eastern Mediterranean Region (Varied)	EMR	Editorial	To highlight the contribution of the Global Health Development (GHD)/Eastern Mediterranean Public Health Network (EMPHNET) and the EMR's Field Epidemiology Training Program (FETPs) (prepare for COVID-19 threat).	The relevant regions and organisations
Ballard et al. 2020 (73)	Not specified	N/A	Nominal group technique	To advocate for rapid action in response to the evolving COVID-19 pandemic	The Community Health Impact Coalition (CHIC)

Costantino & Fiacchini 2020 (58)	Italy (HIC)	EUR	Letter to the editor	To explain the rationale of the WHO interim guidance on Risk Communication and Community Engagement (RCCE) readiness and response to the Severe Acute Respiratory Syndrome Coronavirus 2 (SARS-CoV-2) and of the Italian Decalogue for Prevention Departments.	None
Ippolito et al. 2020 (25)	Europe (HIC)	EUR	Editorial	To contribute to the development of robust common reference systems, coordinate national preparedness plans	None
Lee et al. 2020 (30)	Taiwan (HIC)	None	Commentary	To comment on Taiwan's legal preparedness as part of initial COVID-19 response	None
Subba et al. 2021 (40)	India (LMIC)	SEAR	Editorial	To discuss a conceptual framework for health systems preparedness and response at the level of primary health care institutions (PHCIs)	None

**Ebola virus disease (EVD)**					

Author(s) and publication year	Country under study (World Bank classification)	WHO region	Study design (publication type)	Purpose (abbreviated summary)	Description of study participants for primary studies or data sources for secondary studies
**PRIMARY STUDIES**					
Aceng et al. 2020 (19)	Uganda (LMIC)	AFR	Descriptive report	Reports activities that were undertaken by MoH and its partners from national to health facility levels to enable readiness to effective response	N/A
Espinal et al. 2016 (75)	Latin America and the Caribbean (HIC)	AMR	Descriptive report	Public Health System preparedness frameworks and evaluations for Ebola	N/A
Nanziri et al. 2020 (89)	Uganda (LMIC)	AFR	Descriptive report	Outbreak in the Democratic Republic of the Congo (DRC) preparedness assessment	N/A
Nsubuga et al. 2021 (83)	Uganda (LMIC)	AFR	Mixed methods	Evaluation of the Ebola Virus Disease (EVD) preparedness and readiness program in Uganda that was triggered by outbreak of EVD in neighbouring DRC.	40 key informants from five category 1 districts<br>Qualitative interviews conducted with 32 (91%) participants.
Nyenswah et al. 2016 (32)	Liberia (LIC)	AFR	Descriptive report	Lessons learnt from Liberia's response to EVD with aspects relevant to readiness.	N/A

Otu et al. 2018 (92)	Nigeria (LMIC)	AFR	Qualitative	Lessons learnt from Nigeria's response to EVD including readiness aspects.	Fifteen individuals (10 males and 5 females, ages ranging from 35 to 65 years)
Swaan et al. 2018 (88)	Netherlands (HIC)	EUR	Mixed methods	Article refers to preparedness but describes activities triggered by EVD outbreak in Guinea, Liberia, and Sierra Leone.	Data from Centre for Infectious Disease Control (CID) records<br>48 individuals from 42
Wan et al. 2014 (53)	Malaysia (UMIC)	WPR	Descriptive report (Journal article)	National preparedness in responding to potential EVD importation into Malaysia	N/A

**Pandemic influenza**					
**Author(s) & publication year**	**Country under study (World Bank classification)**	**WHO region**	**Study design (publication type)**	**Purpose (abbreviated summary)**	**Description of study participants for primary studies or data sources for secondary studies**
**PRIMARY STUDIES**					
Itzwerth et al. 2018 (57)	Australia (HIC)	WPR	Document analysis	An analysis of 2009 Influenza pandemic preparedness plans.	10 Australian preparedness plans selected from Australian and State Government sources in print, on official websites, internet resources
Lee 2013 (20)	Republic of Korea (HIC)	WPR	Descriptive report	Comments on attention and caution phases of national disaster in the context of Influenza 2009	N/A

Martin et al. 2010 (82)	All European Union countries and the neighbouring countries of Croatia, Turkey, Iceland, Liechtenstein, and Norway (HIC and one UMIC (Turkey)).	EUR	Cross-sectional survey	Analysis of public health laws framed around an emergent influenza pandemic scenario	The questionnaire was completed by participants with legal and public health expertise from 23 states. Twenty-four countries were represented at review workshops (Czech Republic added).
Stoto et al. 2013 (85)	The USA (HIC)	AMR	Qualitative	Lessons learnt from USA's public health response to 2009 H1N1 Influenza	Purposefully selected participants from a variety of state and local health departments that had prepared H1N1
Tay et al. 2010 (33)	Singapore (HIC)	WPR	Descriptive report	Public health control measures instituted in Singapore (H1N1-2009)	N/A
**MULTIMETHOD STUDIES**					
Hanvoravongchai et al. 2010 (23)	Cambodia, Indonesia, Lao PDR, Taiwan, Thailand, and Viet Nam (Varied)	WPR, SEAR (Taiwan excluded)	Situational analysis: literature review and qualitative	Reports on different stages of pandemic preparedness in relation to health systems context in the six countries.	Secondary data and documentation were reviewed and summarised.<br>21 key informants were selected based on their expertise in a broad range of health system and pandemic programme components.
**OPINION STUDIES**					

Wijesinghe et al. 2020 (35)	WHO South-East Asia Region (Varied)	SEAR	Perspective	Lessons learnt from the implementation of the pandemic influenza preparedness framework	None

**Viral Haemorrhagic Fevers (VHF)**					
**Author(s) & publication year**	**Country under study (World Bank classification)**	**WHO region**	**Study design (publication type)**	**Purpose (abbreviated summary)**	**Description of study participants for primary studies or data sources for secondary studies**
**PRIMARY STUDIES**					
Khan et al. 2018 (22)	Canada (HIC)	AMR	Qualitative	The framework for emergency preparedness pertains to all aspects of emergency management	130 participants from anglophone and francophone communities Focus group participants were practitioners from public health and related sectors.
Khan et al. 2019 (28)	Canada (HIC)	AMR	Modified Delphi technique	The 67 indicators represent important and actionable dimensions of public health emergency preparedness (PHEP) practice	33 experts representing senior-level positions spanning all jurisdictional levels across 12 provinces and territories.
Malik et al. 2013 (38)	Eastern Mediterranean Region (Varied)	EMR	Descriptive report	Report from a technical consultation meeting by the WHO EMR Office with a group of experts to define intervention strategies and public health approaches for	Public health representatives from the countries that are frequently affected by viral haemorrhagic fever (VHF) outbreaks in the Region and a group of WHO experts

				prevention and control of VHF outbreaks in the Region.	
**SECONDARY STUDIES**					
Khan et al. 2015 (26)	Not specified	N/A	Scoping review	Articles included in the scoping review covered public health actions in some aspect of emergency management.	58 articles selected from multiple databases of indexed and grey literature searched based on concepts of public health, emergency, emergency management/preparedness and evaluation/evidence.
**OPINION STUDIES**					
Gibson JP., Theadore & Jellison 2012 (27)	Not specified	N/A	Framework development	The Common Ground Preparedness Framework can be used in response planning.	The 6 grantees each provided 2 or 3 core participants, drawing from 4 state and 4 local health agencies, and occasionally brought in additional experts on subject matter.

AFR- African Region, AMR- Region of the Americas, EMR- Eastern Mediterranean Region, EUR- European Region, HIC- high income country, LIC- low-income country, LIMC- lower middle-income country, SEAR- South-East Asian Region, WHO-World Health Organisation, WPR- Western Pacific Region, U.K.- United Kingdom, UMIC- upper middle-income country, USA- United States of America
