# Supplementary material for: Defining and identifying the critical elements of operational readiness for public health emergency events: a rapid scoping review
Source: BMJ Glob Health. 2024 Aug 29;9(8):e014379. doi: 10.1136/bmjgh-2023-014379 (PMC11367384; doi:10.1136/bmjgh-2023-014379)
Supplement: online supplemental file 4 [file bmjgh-9-8-s004.pdf]

Table 1-B: Characteristics of grey literature publications on the definitions of operational readiness according to emergency type.

**Coronavirus disease 2019**					
**Author(s) and publication year**	**Author affiliations**	**Country under study (World Bank classification)**	**WHO region**	**Document/report type**	**Purpose (Abbreviated)**
Centre for Global Development. 2020 (93)	Centre for Global Development.	United States (HIC)	AMR	**Recommendations** in event that COVID-19 cannot be contained, and sustained human-to-human transmission occurs beyond China.	This paper recommends actions to address pressing gaps in US and global preparedness
Inter-Agency Standing Committee. April 2020 (47)	Inter-Agency Standing Committee	N/A	N/A	**Short technical step-by-step guide** to address the potential non-health impacts of the COVID-19 pandemic	The interim Emergency Response Preparedness (ERP) guidance is designed to be a short technical step-by-step guide aimed at non- Humanitarian Response Plan (HRP) countries
Inter-Agency Standing Committee. May 2020 (52)	Inter-Agency Standing Committee	N/A	N/A	**Interim guidance** to reduce the risk of COVID-19	This Interim Guidance outlines key public health and social measures (COVID-19)
International Organization for Migration. March 2020 (56)	International Organization for Migration (IOM)	N/A	N/A	IOM's COVID-19 global **strategic preparedness and response plan**	Contribute to global, regional and national preparedness and response efforts for COVID-19

International Organization for Migration. April 2020 (55)	International Organization for Migration	Iraq (HIC)	EMR	WHO Iraq Country **Strategic Preparedness and Response Plan**	IOM to contribute to the preparedness, response and recovery in Iraq (COVID-19).
International Organization for Migration. May 2020 (65)	International Organization for Migration	South America (UMIC)	AMR	**Strategic preparedness and response plan** for the region of South America	In view of COVID-19, IOM's regional COVID-19 response for South America
International Organization for Migration. May 2020 (81)	International Organization for Migration	Middle East & North Africa (LMIC)	EMR	**Strategic preparedness & response plan**	IOM, as part of the Inter-Agency Standing Committee, and in partnership with WHO, United Nations Organizations and other stakeholders assisting (COVID-19)
Regional Office for Eastern Mediterranean, World Health Organization. 2020 (91)	WHO Regional Office for the Eastern Mediterranean	Eastern Mediterranean Region (HIC)	EMR	**Regional plan of action** to support the countries of the Region to rapidly accelerate the scaling up of their capacities for the prevention and early detection of, and rapid response to COVID-19	To establish a regional plan of action to support the countries of the Region (COVID-19.
United Nations Office for the Coordination of Humanitarian Affairs. 2020 (44)	United Nations Office for the Coordination of Humanitarian Affairs	N/A	N/A	The COVID-19 Global HRP is a comprehensive inter-agency **response plan**	The Plan focuses on preparedness and response to the initial immediate and urgent health and non-health needs and response to the pandemic, including to secure supply chains and humanitarian personnel mobility.

World Health Organization. February 2020 (64)	World Health Organization	N/A	N/A	**Strategic Preparedness Response Plan** (SPRP) for COVID-19	The SPRP outlines the public health measures that need to be taken to support countries to prepare for and respond to COVID-19.
World Health Organization. 2020 (72)	World Health Organization.	African Region (LMIC)<br>America Region (HIC)<br>Eastern Mediterranean Region (HIC)<br>European Region (HIC)<br>South-East Asia Region (LMIC)	AFR<br>AMR<br>EMR<br>EUR<br>SEAR	**Progress report –** highlights the main points of progress that were made up to 30 June 2020 under the three objectives outlined in the SPRP for COVID-19	This report highlights the main points of progress that were made up to 30 June 2020 under the three objectives outlined in the SPRP.
World Health Organization. 2020 (62)	World Health Organization.	N/A	N/A	Covers key areas unique to urban settings, **supplements** other COVID-19 documents, including the WHO SPRP and the strategy update	This document aims to support local authorities, leaders and policymakers in cities and other urban settlements in identifying effective approaches – taking into consideration urban vulnerabilities
World Health Organization. 2021 (61)	World Health Organization	N/A	N/A	**Guidance document** – COVID-19 SPRP 2021 is intended to help guide the public health response to COVID-19	The COVID-19 SPRP 2021 is intended to help guide the public health response to COVID-19 at national and subnational levels, and to update the global strategic priorities in support of this effort.

World Health Organization Regional Office for Europe. 2020 (90)	World Health Organization.	European Region (HIC)	EUR	**Checklist –** to ensure that countries are ready at the local and national levels – COVID-19	The main aim of this checklist is to ensure that countries are ready at the local and national levels to detect sick people, test samples of those suspected of COVID-19, manage patients adequately, maximise infection control, and maintain open communication with the public.

**Influenza pandemic**					
**Author(s) and publication year**	**Author affiliations**	**Country under study (World Bank classification)**	**WHO region**	**Document/report type**	**Aim/purpose**
World Health Organization. 2018 (59)	World Health Organization.	Global	N/A	The Pandemic Influenza Preparedness **Framework** is an international arrangement adopted by the World Health Assembly in May 2011 to improve global pandemic influenza preparedness and response	It aims to improve the sharing of influenza viruses with pandemic potential (IVPP), on the one hand, and the equitable access to products necessary to respond to pandemic influenza (e.g., vaccines, antiviral medicines, and diagnostic products), on the other. Under the Framework, implementation strives to build sustainable capacities for detecting and responding to pandemic influenza.

**Unspecified hazards/incidents**					
**Author(s) and publication year**	**Author affiliations**	**Country under study**	**WHO region**	**Document/report type**	**Aim/purpose**

		(World Bank classification)			
Centres for Disease Control and Prevention. 2011 (45)	Centres for Disease Control and Prevention.	United States (HIC)	AMR	A National strategic plan for public health preparedness and response	The strategic priorities described are a guide for CDCs, the entire public health system, and its stakeholders to secure the health of our nation.
Centres for Disease Control and Prevention. 2018 (95)	Centres for Disease Control and Prevention.	United States (HIC)	AMR	Public health emergency preparedness and response capabilities model	The National Preparedness System outlines an organised process for everyone in the whole community to advance their preparedness activities and achieve the National Preparedness Goal.
Centres for Disease Control and Prevention. 2021 (94)	Centres for Disease Control and Prevention.	CDC Recipients	N/A	CDC's Operational Readiness **Review**	CDC's Operational Readiness Review is a rigorous, evidence-based assessment used to evaluate PHEP program planning and operational functions.
Regional Office for Europe, World Health Organization. 2019 (60)	World Health Organization	Europe (HIC)	EUR	**Brochure** – part of a toolbox for WHO Country Offices to encourage ministers of health to act; for ministers of health to engage their governments to act; and for governments to persuade donors and the international community to act	WHO working with countries in the European Region - all types of health emergencies
World Health Organization. 2013 (86)	World Health Organization	N/A	N/A	Emergency Response **Framework**	Emergency Response Framework to clarify WHO's roles and responsibilities in emergency response and to provide a common approach.

World Health Organization. 2018 (68)	World Health Organization.	N/A	N/A	**Contingency planning guidance**, to prepare for emergencies from all hazards.	Contingency planning guidance, a set of actions to prepare for emergencies from all hazards and to help minimise their impact, is proposed.
World Health Organization. 2018 (18)	World Health Organization.	N/A	N/A	A strategic regional plan for preparedness and operational readiness in countries bordering the Democratic Republic of Congo against EVD	To ensure that the identified high risk countries are operationally ready to detect and respond to a potential importation of a new case, prepared to detect and manage cases beyond the initial three months, ensure sustainable preparedness, strengthen IHR core capacities to manage outbreaks and other health emergencies, and build resilient health systems.
World Health Organization. 2019 (34)	World Health Organization	N/A	N/A	The Health Emergency and Disaster Risk Management (EDRM) **Framework** provides an overview of risk management concepts, guiding principles, the components and functions of effective Health EDRM, and guidance on implementing the framework	Document to provide ministries of health and other stakeholders with a summary of policy considerations to reduce the risks and consequences of emergencies and disasters, and build the resilience of health systems, communities, and countries.
World Health Organization. 2021 (74)	World Health Organization.	N/A	N/A	The **framewor**k and **toolkit** described target the prevention and control of communicable diseases with community outbreak potential	A practical framework of actions for strengthening IPC outbreak preparation, readiness, and response; and a toolkit that provides resources to assist in the development of local contingency or action plans to strengthen IPC outbreak preparedness, readiness, and response.

World Health Organization. 2021 (84)	World Health Organization	N/A	N/A	**Framework** for developing capacities for International Health Regulations (IHR), and components in health systems and other sectors that work in synergy to meet the demands imposed by health emergencies	'Health systems for health security' framework to support countries, WHO and partners.
