# Supplementary material for: Defining and identifying the critical elements of operational readiness for public health emergency events: a rapid scoping review
Source: BMJ Glob Health. 2024 Aug 29;9(8):e014379. doi: 10.1136/bmjgh-2023-014379 (PMC11367384; doi:10.1136/bmjgh-2023-014379)
Supplement: online supplemental file 5 [file bmjgh-9-8-s005.pdf]

Supplemental Table S2: Study characteristics and readiness/preparedness definitions

Author(s) & publication year	Level (community, national, etc.)	Type of emergency	Preparedness definition<br>Readiness definition	Key actors
**PRIMARY STUDIES**				
Aceng et al. 2020 (19)	National and health facility	Ebola virus disease (EVD)	The epidemic preparedness process constituted all the activities that were undertaken by Ministry of Health and its partners from national to health facility levels to enable readiness to effective response to EVD outbreak in Uganda from August 2018 when EVD outbreak was declared in the DRC to the time of writing.	Ministry of Health, Public Health Emergency Operations Centre (PHEOC) personnel and the National Task Force (NTF) for public health emergencies
Bennett 2021 (51)	National and state public health	COVID-19	Not mentioned.	Australian Health Protection Principal Committee (AHPPC): the Communicable Disease Network of Australia (CDNA), the Public Health Laboratory Network (PHLN), and the Infection Control Expert Group (ICEG)
Espinal et al. 2016 (75)	National	Ebola virus disease (EVD)	Not mentioned.	The Pan American Health Organisation (PAHO) and partner agencies
Khan et al. 2018 (22)	Local/ regional	Not specified†	Used the WHO definition of emergency preparedness: '…the knowledge and capacities and organisational systems developed by governments, response and recovery organisations, communities and individuals to effectively anticipate, respond to, and recover from the impacts of likely, imminent, emerging, or current emergencies'.	Local/ regional public health agencies
Khan et al. 2019 (28)	Local/ regional	Not specified†	Not mentioned.	Local/ regional public health agencies
Lee 2013 (20)	National	Pandemic influenza	Preparedness is a series of activities in planning, preparation, education, and training to enhance the capabilities of public health personnel.	Division of public health crisis response in Korea Centers for Disease Control and Prevention
Liu & Saltman 2020 (66)	National	COVID-19	Not mentioned.	Chinese government: central, provincial, and municipal.
Malik et al. 2013 (38)	Regional and national	Viral haemorrhagic fevers (VHF)	Not mentioned.	WHO and public health representatives
Martin et al. 2010 (82)	Regional and national	Influenza H1N1 virus	Not mentioned.	Public health policy makers and pandemic planners (legal experts)
Moonasar et al. 2021 (37)	National	COVID-19	Not mentioned.	Ministry of Health, national and provincial Incident Management Teams
Nanziri et al. 2020 (89)	District	Ebola virus disease (EVD)	Not mentioned.	Ministry of Health, public health emergency management personnel and the National Task Force
Neupane et al. 2020 (87)	National	COVID-19	Not mentioned.	Ministry of Health and Population (MoHP)
Nguyen et al. 2021 (71)	National	COVID-19	Not mentioned.	Government, Ministry of Health, health systems and media

Nsubuga et al. 2021 (83)	National and district	Ebola virus disease (EVD)	Not mentioned.	Ministry of Health, the national and district task force, and village health teams
Nyenswah et al. 2016 (32)	National	Ebola virus disease (EVD)	Not mentioned.	Ministry of Health and Social Welfare (MOHSW)
Oh et al. 2020 (36)	National	COVID-19	Not mentioned.	The Ministry of Health and Welfare and the President's Office
Oliveira et al. 2020 (79)	National	COVID-19	Not mentioned.	The Ministry of Health
Otu et al. 2018 (90)	National	Ebola virus disease (EVD)	Not mentioned.	Lagos State Government in conjunction with the Federal Ministry of Health
Paudyal et al. 2021 (65)	Clinical services	COVID-19	Not mentioned.	Clinical pharmacists
Stoto et al. 2013 (85)	National and local	2009 H1N1 influenza pandemic	Not mentioned.	State and local public health officials
Swaan et al. 2018 (88)	Regional and national	Ebola virus disease (EVD)	Not mentioned.	Centre for Infectious Disease Control (CID) of the National Institute for Public Health and the Environment (RIVM) coordinates response on a national level on behalf of the Ministry of Health. Public health services (PHSs) coordinate preparedness and response regionally.
Tay et al. 2010 (33)	National	Influenza A (H1N1-2009)	Not mentioned.	Ministry of Health and National Task Force
Tran et al. 2020 (77)	Community	COVID-19	Not mentioned.	District health system policy makers and practitioners
Wan et al. 2014 (53)	National	Ebola virus disease (EVD)	Not mentioned.	The National Crisis Preparedness and Response Centre (CPRC), which is the national command centre for infectious disease surveillance under the Ministry of Health.
**SECONDARY STUDIES**				
Biswas et al. 2020 (54)	National	COVID-19	Not mentioned.	Health system policy makers and practitioners
Ghanbari et al. 2020 (41)	National	COVID-19	N/A	Ministry of Health
GRID Covid Study Group 2020 (31)	National	COVID-19	Not mentioned.	Government of India, state governments and Ministry of Health and Family Welfare (MOHFW).
Hasan et al. 2021 (76)	Health systems	COVID-19	Not mentioned.	Health system policy makers and practitioners
He et al. 2020 (46)	Federal	COVID-19	Not mentioned.	National Security Council (NSC), National Crisis Centre (NCCN), Federal Coordination Committee (COFECO), the Ministers-President of the Regions and Communities, FPS Public Health, Risk Assessment Group (RAG), Risk Management Group (RMG), Scientific Committee for Coronavirus, and some other units

Itzwerth et al. 2018 (57)	National, state, and territorial.	Influenza	Not mentioned.	Pandemic planners
Kandel et al. 2020 (24)	National	COVID-19	Achieving **readiness** is a continuous process of establishing, strengthening, and maintaining a multisectoral response infrastructure that can be applied at all levels, which follows an all-hazard approach, and which focuses on the highest priority risks. Operational readiness builds on existing capacities to design and set up specialised arrangements and services for an emergency response.	Not mentioned
Khan et al. 2015 (26)	National, state/provincial, local	Not specified†	Public health emergency preparedness has been defined as "the capability of the public health and health care systems, communities, and individuals, to prevent, protect against, quickly respond to, and recover from health emergencies, particularly those whose scale, timing, or unpredictability threatens to overwhelm routine capabilities". Examples of actions of PHEP related to health emergencies include surveillance and epidemiologic activities to monitor, detect, and investigate potential health threats, and the development and communication of information to the public.	Public health and health systems policy makers and practitioners and communities.
Mohammadpour et al. 2021 (70)	Health systems	SARS, MERS, and COVID-19	Not mentioned.	Government and health system policy makers and practitioners
Neogi & Preetha 2020 (29)	Health systems	COVID-19	Not mentioned.	N/A
Petrović et al. 2020 (21)	National	COVID-19	**Structural readiness**: reflects the state in which society awaits the pandemic. It includes three dimensions: health system preparedness, trust in institutions, and health risk factors.<br>Preparedness is a country's capacity to deal with the importation and the spread of the virus.	Government and health system policy makers
Raoofi et al. 2020 (48)	National (health policy)	COVID-19	Not mentioned.	Actors include a wide range of governmental and non- governmental organisations, interest groups, media, religious groups, etc, that influence various stages of the policy process. The Ministry of Health and Medical Education (MoHME), as the stewardship of health, is the key actor in the fight against COVID-19 in Iran. The Universities of Medical Sciences (UMS), the MoHME various deputies (particularly Deputies for Curative Affairs, Public Health, Nursing, and Administration and Finance), Food and Drug Organisation, Emergency Services Organisation, and Iran Health Insurance Organisation are the main intra-organisational actors within the MoHME, plus almost 200 Medical Associations, General Medical Council, General Nursing Council, Social Security Organisation (and other insurance organisations), are also among main actors in policymaking, implementation and evaluation of related policies.

Santos et al. 2021 (43)	National, state, and federal district.	COVID-19	Not mentioned.	Pandemic planners
Shimizu & Negita 2020 (49)	National	COVID-19	Not mentioned.	Ministry of Health, Labour, and Welfare and government
Simões et al. 2020 (42)	National	COVID-19	Not mentioned.	Government and health system policy makers and practitioners
Sohrabizadeh et al. 2021 (39)	Health systems	Earthquake, flood, hurricane, tornado, or wildfire + COVID-19	Not mentioned.	Health system policy makers and practitioners
Wang et al. 2020 (80)	National	COVID-19	Not mentioned.	Government and community
Wang et al. 2020 (69)	National	COVID-19	Not mentioned.	Health system policy makers and practitioners
Yang et al. 2021 (63)	National	COVID-19	Not mentioned.	Government and health system policy makers and practitioners
**MULTIMETHOD STUDIES**				
Kapiriri et al. 2021 (50)	National	COVID-19	Not mentioned.	Ministry of Health, health system policy makers and practitioners
Hanvoravongchai et al. 2010 (23)	Health systems	H1N1/2009	Preparedness is a complex phenomenon which involves many aspects, including disease surveillance, case management, command and control, and community containment.	Health system policy makers and practitioners
Seyedin et al. 2021 (96)	National and hospital	COVID-19	Not mentioned.	Ministry of Health and hospitals
**OPINION STUDIES**				
Al Nsour et al. 2020 (76)	Regional and national	COVID-19	Not mentioned.	Global Health Development (GHD)/ Eastern Mediterranean Public Health Network (EMPHNET)
Ballard et al. 2020 (73)	Community	COVID-19	Not mentioned.	Ministries of Health, regional authorities, and communities.
Costantino & Fiacchini 2020 (58)	National	COVID-19	Not mentioned.	The Working Group for Communication in Public Health of the Italian Society of Hygiene, Preventive Medicine and Public Health (the main Italian Scientific Society for Public Health professionals)
Gibson, Theadore. & Jellison 2012 (27)	National and community	Not specified†	The capability of the public health and health care systems, communities, and individuals, to prevent, protect against, quickly respond to, and recover from health emergencies, particularly those whose scale, timing, or unpredictability threatens to overwhelm routine capabilities.	Public health agencies

Ippolito et al. 2020 (25)	National and community	COVID-19	Preparedness should include upstream prevention through better collaboration of different sciences, to enhance capacity to identify potential pathogens before they become serious human threats, and to prevent their emergence where possible.	Health system policy makers and practitioners
Lee et al. 2020 (30)	National	COVID-19	Not mentioned.	Government
Subba et al. 2021 (40)	Primary health care system	COVID-19	Not mentioned.	Primary health care institutions (PHCIs)
Wijesinghe et al. 2020 (35)	Regional	Pandemic influenza	Not mentioned.	Member States and WHO
Grey literature				
World Health Organization. 2018 (18)	Regional	EVD	**Operational Readiness** includes being operationally ready to detect and respond to a potential importation of a new case. It takes place in a broader context of emergency preparedness and therefore also responds to the needs of countries to build sustainable capacities to manage outbreaks and other health emergencies.	Member States and WHO

AHPPC, Australian Health Protection Principal Committee; CDNA, Communicable Disease Network of Australia; CID, Centre for Infectious Disease Control; COFECO, Federal Coordination Committee; COVID-19, Coronavirus Disease 2019; CPRC, Crisis Preparedness and Response Centre; DRC, Democratic Republic of the Congo; EMPHNET, Eastern Mediterranean Public Health Network; EVD, Ebola virus disease; GHD, Global Health Development; ICEG, Infection Control Expert Group; MERS, Middle East respiratory syndrome; MoHP, Ministry of Health and Population; MOHSW, Ministry of Health and Social Welfare; N/A, not applicable; NCCN, National Crisis Centre; NSC, National Security Council; NTF, National Task Force; PAHO, Pan American Health Organisation; PHCIs, primary health care institutions; PHEOC, Ministry of Health, Public Health Emergency Operations Centre; PHLN, Public Health Laboratory Network; PHSs, Public health services; RAG, Risk Assessment Group; RMG, Risk Management Group; SARS, severe acute respiratory syndrome; UMS, Universities of Medical Sciences; WHO, World Health Organisation.

Note: Information in the table above is verbatim from the respective documents.
